# Adventitious Bursae Underlying Chronic Wounds: Another Possible Deterrent to Healing

**Published:** 2012-02-23

**Authors:** Burkley Jensen, Brian Leykum, Joseph Fiorito, Darren Woodruff, Manish Bharara, David G. Armstrong

**Affiliations:** ^a^Midwestern University School of Podiatric Medicine, Glendale, AZ; ^b^University of Arizona Health Sciences Center, Tucson, AZ

## Abstract

Adventitious bursae typically develop in areas of chronic frictional irritation, usually under bony prominences. Although adventitious bursae are generally well understood, there is a paucity of data on effects of bursae underlying chronic wounds in neuropathic patients. This manuscripts reviews 4 clinical cases, each with a neuropathic patient with adventitious bursae underlying chronic nonhealing wound and strategies for treatment.

The Wound Healing Society defines *chronic ulcerations* as wounds that have “failed to proceed through an orderly and timely process to produce anatomic and functional integrity, or proceeded through the repair process without establishing a sustained anatomic and functional result.”^1^ Chronic wounds resulting in indolent ulcers are similar in that each is characterized by 1 or more persistent inflammatory stimuli: repeat trauma, ischemia, or bacterial contamination.^2^ These wounds place a great burden both on the individual and on the health care system, with the conservative cost of treatment estimated at $20 billion per year in the United States alone.^3^ It has been the experience of the authors of this article that adventitious bursae formation has been found to be another contributing source to delaying the healing process of a chronic foot wound when all other measures to promote healing were accounted for.

## ADVENTITIOUS BURSAE

There are 2 different types of bursae that have been described; these are synovial bursa and adventitious bursa. Synovial bursae are called such as they are synovial-lined structures that function to decrease friction and shear between 2 moving structures.^4^ These bursae develop during weeks 20 to 40 of development over areas where some degree of motion is required between skin, tendon, and bony structures. Adventitious bursae develop postpartum and arise from friction and pressure in superficial fibrous connective tissue in locations where the skin must move freely over subcutaneous bony surfaces.^5^^,^^6^

There is some disagreement over the contents of adventitious bursae. There are several views: one is that recurrent stress breaks down the matrix of supporting tissues leading to focal fluid collection,^7^ another view is that they are a cystic structure filled with cellular debris, extracellular fluid, altered ground substance, and inflammatory exudate,^8^ and the original view of Virchow is that “the bursae are in no sense true serous sacs but, are rather places where the connective tissue, originally present ‘in continuo,’ forms spaces by a process of atrophy and where these spaces come in time to be independent cavities.”^9^ (p126)

The foot is a myriad of moving parts bound together in a compact space and when subjected to stress and strain the foot is a fertile field in which adventitious bursa can develop.^10^ There have been several articles published in which nonanatomic bursae have been noted in the feet on the medial aspect of the first metatarsophalangeal joint when a hallux abducto valgus deformity is present.^10^^,^^11^ Adventitious bursae have also been noted underlying new bony prominences in neuropathic patients with Charcot deformities, but little has been published on this subject. Through a comprehensive literature search, we have found one case report mentioning intractable malleolar bursitis impairing the healing of surgical incisions. This report contained 2 case studies of dehisced incision sites and the surgical team had to resect the bursa in order to obtain wound healing.^12^ To our knowledge, there are no descriptions of adventitious bursae underlying chronic wounds on the feet in neuropathic patients in the peer-reviewed literature. For this reason, the purpose of this article is to describe this malady by way of several case studies of patients with adventitious bursae impairing healing of neuropathic wounds.

### Case 1

**Figure 1:**
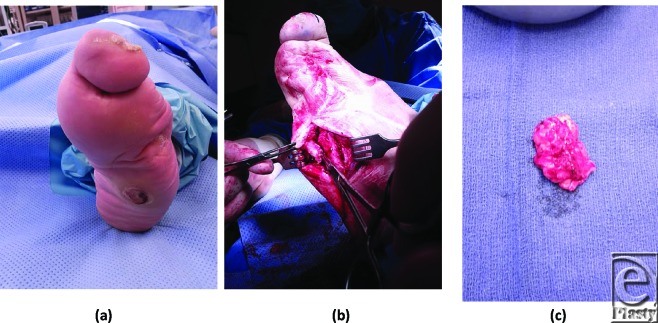
Adventitious bursa underlying neuropathic wound on lateral column of foot.

A 57-year-old man with a history of diabetes mellitus type 2 and hypertension was referred to our clinic with a chronic ulceration on the plantar lateral aspect of the left foot. The patient had an amputation of digits 2 to 5 on the left foot due to an infection a few years prior. As a consequence of the amputation, the patient had a contracted and lateral deviated left hallux. Subsequently, the patient developed a blister around the plantar base of the remaining fifth metatarsal, which progressed to an ulceration. Physical examination revealed a 2-cm^2^ ulcer with a granular base and a rim of hyperkeratosis. Clinical signs of infection were absent. Upon palpation and radiographic evaluation, a prominent fifth metatarsal head was discovered deep to the ulcer. The wound was treated with Medi-honey (Derma Sciences, Princeton, NJ) and dressing changes were performed by home health. The patient was placed in custom molded offloading footgear. The patient followed up in clinic every other week, but the wound failed to heal. Surgical debridement of the bony prominence was scheduled. Intraoperatively, a bursa deep to the ulceration was discovered, and the specimen was sent for pathology. Surgical pathology revealed fibrous and bursal tissue (Fig 1).

### Case 2

**Figure 2:**
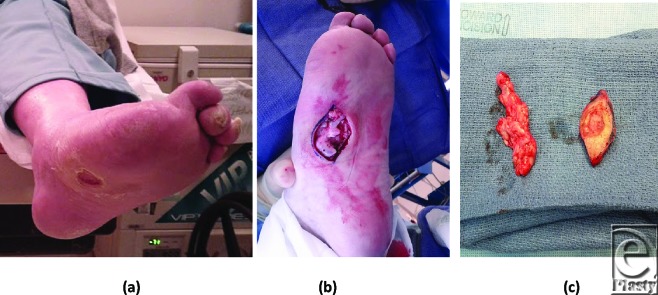
Wound with bursa under midfoot following skin grafting and near complete healing.

A 76-year-old woman with a history of idiopathic neuropathy who had previously underwent a right trans-metatarsal amputation and Charcot foot reconstruction with application of external fixation device. A total contact was applied weekly for 3 weeks after removal of the external fixation device to the right leg. Two weeks after removal of the third total contact cast, the patient presented to the clinic with a new ulceration on the medial plantar aspect of the left mid-foot measuring 3 cm^2^. The ulcer presented with a hyperkeratotic rim and a granular base. The wound did not probe to bone, and there was no purulence, fluctuance, or other clinical signs of infection. Over the course of the next 2 months, 6 serial bioengineered grafts were applied to the ulceration site following debridement resulting in only minor reduction in wound size. Surgical debridement was, therefore, scheduled. Intraoperatively, a bursa deep to the ulceration was readily identified and the specimen sent for pathology. Results from pathology revealed chronic inflammation, bursal tissue, and fibrosis (Fig 2).

### Case 3

**Figure 3:**
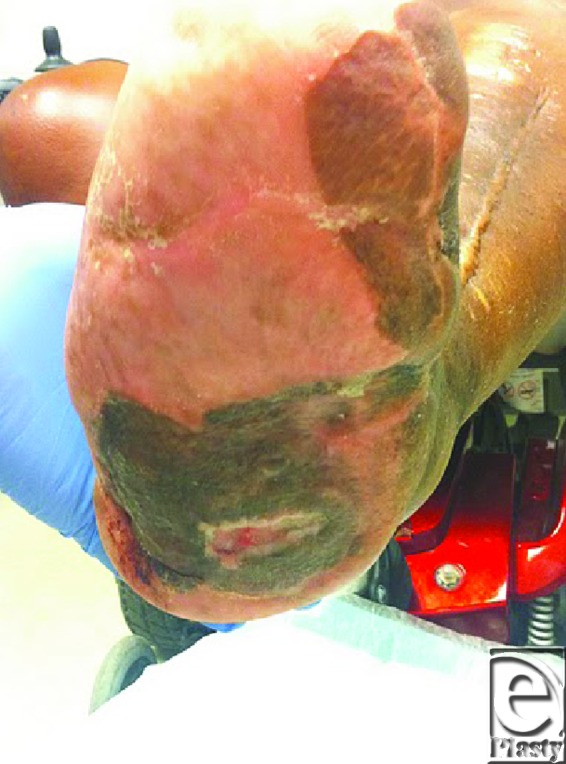
Wound with bursa under midfoot following skin grafting and near complete healing.

A 67-year-old woman with a history of diabetes mellitus type 2 and Charcot deformity on her right foot presented with a nonhealing ulcer on the plantar aspect of her right mid-foot. She was involved in a randomized parallel group double-blind placebo-controlled phase 2 clinical trial to evaluate the effectiveness of DSC 127 in treating subjects with diabetic ulcers. She was placed in a long cam-walker for the duration of the trial, which was 4 months. Her ulcer size at the beginning of the trial was 1.9 × 0.6 × 0.2 cm and at the end was 1.7 × 0.5 × 0.2 cm. Because of the minimal healing during the trial, surgical debridement of soft tissue and the underlying bony exostosis were scheduled. Intraoperatively, a bursa deep to the ulceration was discovered (Fig 3).

### Case 4

**Figure 4:**
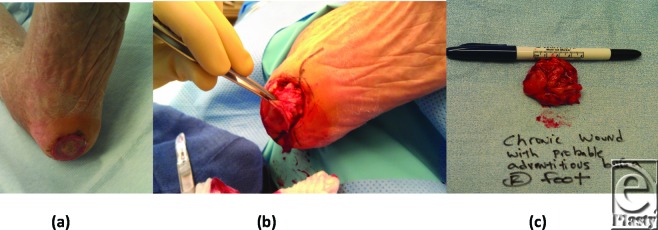
Neuropathic plantar heel wound with underlying adventitious bursa.

A 82-year-old woman with a history of diabetes mellitus type 2, hypertension, and previous Achilles tendon rupture, which resulted in a calcaneal gait, presented with a nonhealing ulcer to her left heel. The patient was entered into an offloading study on total contact casting that failed to heal the wound. Over the course of the following 2 months, the patient was treated with the application of 8 serial bioengineered grafts, while being offloaded in a DH walker, but the wound showed little improvement. Because of failure of conservative treatment, surgical intervention was indicated. The patient was brought to the operating room at which time the wound was excised and a bursa deep to the ulceration was discovered. A partial calcanectomy and anterior tibial Tenotomy were performed at the same time. Results from pathology revealed fibrotic and cystic tissue (Fig 4).

## DISCUSSION

The manuscript presents cases highlighting the potential clinical importance of adventitious bursae deep to chronic ulcers. To our knowledge, this is the first report in the medical literature describing this phenomenon. It is likely, however, that the paucity of reports belies a higher prevalence in actual practice. In reviewing the cases presented, all of the patients were neuropathic and had developed foot deformities secondary to previous amputation or Charcot. We postulate that these bursa and ulcerations are occurring on areas that were previously non–weight-bearing surfaces but, through deformity, have come to be weight-bearing areas subject to excessive friction and pressure. This has been reported in the literature to happen with below-the-knee amputees when muscle or other redundant soft tissue was folded over the distal aspect of the amputation.^13^ It is of interest whether the same process happens with other more distal amputations.

Another area of interest is whether these bursae are preventing the ulcerations from closing. It may be argued that the underlying bursae are blocking new vessel growth or a chronic inflammatory reaction stalling the healing cascade. An adventitious bursa may also prevent surrounding tissue from healing around it as it is a space-filling lesion. On the contrary, perhaps the bursae are ineffective in their purpose to control shear; thus, there is continued breakdown of skin leading to ulceration.

## CONCLUSION

An understanding of the true etiology of adventitious bursae and their association with chronic ulceration will enhance prevention and treatment of these maladies. It is unclear at this point in time whether these bursae are truly advantageous and are reducing shear or whether they are in some unknown mechanism preventing closure of chronic ulcerations. Further investigation is needed on this subject, as this case series is limited because of the lack of literature and documented cases.
